# The digital twin in neuroscience: from theory to tailored therapy

**DOI:** 10.3389/fnins.2024.1454856

**Published:** 2024-09-17

**Authors:** Lucius Samo Fekonja, Robert Schenk, Emily Schröder, Rosario Tomasello, Samo Tomšič, Thomas Picht

**Affiliations:** ^1^Cluster of Excellence Matters of Activity, Image Space Material, Humboldt-Universität zu Berlin, Berlin, Germany; ^2^Department of Neurosurgery, Charité - Universitätsmedizin Berlin, Berlin, Germany; ^3^Brain Language Laboratory, Department of Philosophy and Humanities, Freie Universität Berlin, Berlin, Germany; ^4^University of Fine Arts of Hamburg, Hamburg, Germany

**Keywords:** digital twin, tumor, network neuroscience, simulation, translational medicine, philosophy, plasticity, theory

## Abstract

Digital twins enable simulation, comprehensive analysis and predictions, as virtual representations of physical systems. They are also finding increasing interest and application in the healthcare sector, with a particular focus on digital twins of the brain. We discuss how digital twins in neuroscience enable the modeling of brain functions and pathology as they offer an in-silico approach to studying the brain and illustrating the complex relationships between brain network dynamics and related functions. To showcase the capabilities of digital twinning in neuroscience we demonstrate how the impact of brain tumors on the brain’s physical structures and functioning can be modeled in relation to the philosophical concept of plasticity. Against this technically derived backdrop, which assumes that the brain’s nonlinear behavior toward improvement and repair can be modeled and predicted based on MRI data, we further explore the philosophical insights of Catherine Malabou. Malabou emphasizes the brain’s dual capacity for adaptive and destructive plasticity. We will discuss in how far Malabou’s ideas provide a more holistic theoretical framework for understanding how digital twins can model the brain’s response to injury and pathology, embracing Malabou’s concept of both adaptive and destructive plasticity which provides a framework to address such yet incomputable aspects of neuroscience and the sometimes seemingly unfavorable dynamics of neuroplasticity helping to bridge the gap between theoretical research and clinical practice.

## Introduction

Various advancements within the last decade are summarized under the umbrella of the fourth industrial revolution, be it a shift toward automation, data exchange, or the Internet of Things. One notable advancement among these is the emergence of digital twin concepts. At its core, a digital twin is a virtual representation of a physical object, system, or process, often updated with real-time data to mirror the life cycle of its physical counterpart. Such systems are already successfully deployed in manufacturing (Rosen et al., 2015), design (Helbing and Sánchez-Vaquerizo, 2023), architecture (Al-Sehrawy and Kumar, 2021) or city planning (Ravid et al., 2022) among others.

Digital twins are also becoming pivotal tools in healthcare, marking significant progress at the intersection of technology and translational medicine (Woolf, 2008). In the context of healthcare, digital twins may represent anatomical structures or simulate dynamic processes, particularly in disciplines like neuroscience, where they model brain functions and predict clinical outcomes. While the concept is well-established in fields like engineering and manufacturing, its application in neuroscience, particularly in neuro-oncology, is still evolving. This approach focuses on applying basic research findings to clinical studies, that could support the development of new medical solutions. Within the medical field, definitions of digital twins diverge, ranging from simple anatomical representations to complex, and interactive systems that simulate brain dynamics in relation to pathology, stimuli, or interventions with predictive models (Ritter et al., 2013; Shams et al., 2023; Fekonja et al., 2022; Fekonja et al., 2021). Thus, in the medical and neuroscientific disciplines, digital twins embody the interplay between virtual models and biological realities. Their adaptability not only enhances our comprehension of complex physiological phenomena but also enables us to reflect on theoretical concepts underlying phenomena such as aspects of intelligence, consciousness, or plasticity and adaptivity. Hence, digital twins hold additionally the potential to bridge the gap between theoretical research, clinical practice, and basic research within neuroscience. However, challenges remain, including the failure of some models to accurately predict patient-specific outcomes due to the complexity of brain networks and tumor behavior, which highlights the limitations of a purely neuroscientific approach (Jirsa et al., 2017). Addressing these complexities requires a more holistic approach that integrates not only advanced computational and biological models but also philosophical perspectives to better understand and simulate the brain’s adaptive and maladaptive plasticity. This integration is particularly crucial in neuro-oncology, where the unpredictable nature of tumor growth and brain plasticity presents significant challenges to model accuracy and clinical applicability.

Given the complexity and interdisciplinary nature of digital twins, collaboration between model developers, clinical practitioners, and philosophers is needed. This collaborative approach ensures that digital twins are not only grounded in clinical reality but are also informed by rigorous theoretical frameworks. By closely working with clinicians, model builders can refine the inputs to avoid the inclusion of excessive or irrelevant data and ensure that critical biological factors are not overlooked. At the same time, engaging with philosophers, such as Malabou, provides a deeper theoretical foundation, particularly regarding concepts like neuroplasticity, thus enhancing both the clinical applicability and the broader existential and ethical dimensions of these models.

Here, we will focus on brain tumors and demonstrate how digital twins can help us understand the impact of such a pathology on the functioning and materiality of the brain (Salvalaggio et al., 2023; Shams et al., 2022; Fekonja et al., 2022; Tuncer et al., 2022; Fekonja et al., 2021; Tuncer et al., 2021). Moreover, the significant changes induced by brain tumors and their subsequent resection demonstrate the broader concept of plasticity, which encompasses not only adaptive but also maladaptive forms. While destructive plasticity captures these negative changes, the established term in neurology is maladaptive plasticity. We will explore these dimensions and show how digital twins can bridge the gap between theoretical concepts and the practical reality of brain pathologies.

## Digital twins in neuroscience and how they are applied to understand brain tumors

A large body of work in the field of neuroscience is dedicated to understanding the sophisticated and complex interactions between billions of neurons that give rise to human cognition. This research spans multiple levels of structure within the human brain, from molecules and neurons to neural circuits, cortical areas and entire systems that make up the brain (Petersen and Sporns, 2015). The simulations of the structure of the human brain with precise mathematical models offer a framework to simulate cerebral processes of the human brain by digital means. They can facilitate a deeper understanding of neuronal activity underlying cognitive processes of healthy brains as well as advancing our knowledge of neurological conditions, ranging from developmental disorders to neuropathology, such as brain tumors (Tomasello et al., 2024). However, they may as well help understand the complex functioning of a healthy brain. Digital twinning in neuroscience extends beyond simple replication of brain processes; it involves the abstraction and simplification of complex neural activity to create operational models. These models, guided by current technology and our understanding, are developed for specific purposes, such as predicting surgical outcomes or modeling neuronal firing patterns. This approach is necessary due to the complicated nature of brain function and the technological limitations we face. It also entails integrating analyses to predict disease impact, evaluate therapeutic strategies, and tailor treatments to the individual’s specific pathology, causal concepts, and brain reconfigurations (Finc et al., 2020; Srivastava et al., 2022; Ross and Bassett, 2024). Thus, a digital twin emerges not only as a tool of representation but potentially as an interactive, dynamic tool as well for experimentation and discovery.

Digital twins within neurosurgery are currently mainly based on magnetic resonance imaging (MRI) data. The resulting neuroimaging data are composed of voxels, a combination of the words *volume* and *pixel*. Whereas a pixel is a small square unit on a 2-dimensional plane, a voxel is a cube-shaped, 3-dimensional unit. Each voxel contains data about brain structures or activity. Such Activity can be measured through functional MRI (fMRI), which provides a dynamic image by measuring blood flow related to neural activity, thereby providing the opportunity to simulate brain functions and potentially detect patterns associated with specific cognitive tasks or disorders. Diffusion MRI (dMRI), on the other hand, traces water molecule movements in the brain tissue, offering insights into the structural connectivity between brain regions (Jeurissen et al., 2019). One prominent application of these imaging methods in neuroscientific digital twinning is The Virtual Brain (TVB) software (Aerts et al., 2020; Aerts et al., 2018; Ritter et al., 2013). TVB integrates manifold data to construct personalized, mathematical, dynamic brain models on well-established biological principles known to exist to simulate human-specific cognitive functions at the cellular and cortical level and to elucidate, for instance, topographical differences in neural activity responses documented through the various neuroscientific methods mentioned above. Thus, with such tools, we can simulate how brain regions interact and respond to various stimuli, diseases, or even potential neurosurgical interventions, and understand the brain in a controlled virtual environment. Central to a more complex digital twin in neuroscience is the integration of multi-modal data, including above introduced MRI data, but also neuropsychological scores, quality of life assessments and non-invasive brain stimulation findings. This integration thus facilitates the evaluation and modeling of anatomical changes, such as those induced by a tumor, and their effects on the anatomy, network, functionality, and subjective well-being in relation to the individual’s central nervous system.

Brain tumors affect not only the physical structure of the brain but also its functional integrity, challenging medical professionals to find solutions that address both pathological and compensatory mechanisms at play. A digital twin, with its capacity to simulate and predict complex dynamics, offers a unique opportunity to understand and respond to such conditions. By integrating data from neuroimaging, genomic analyses, and clinical outcomes, digital twins can model tumor effects, the surrounding brain tissue’s response, and the efficacy of proposed treatments, marking a new era of personalized therapy.

This personalization requires careful consideration of model selection. Different aspects of digital twins in neuroscience necessitate tailored approaches based on the specific clinical or research question. For instance, while MRI-based models may suffice for structural analysis, functional and network-based approaches might be necessary to simulate cognitive processes or tumor-induced changes. Integrating diverse modeling techniques that align with clinical objectives is essential to enhance the predictive accuracy of digital twins across various scenarios (Aerts et al., 2020).

Moreover, biology-guided model selection, which considers underlying biological mechanisms, is crucial for digital twins in neuroscience (Tomasello et al., 2024; Tomasello et al., 2018; Sarma et al., 2010). Traditional statistical methods might not adequately capture the complexities of brain dynamics, requesting models that integrate biological insights, such as neuroplasticity and cellular responses, to improve the relevance and accuracy of simulations.

Furthermore, digital twin architectures should be adaptable to specific clinical problems. For instance, tumor models require additional components for simulating tumor growth and/or infiltration, while epilepsy models may need more detailed neural mass models to capture seizure dynamics (Shams et al., 2023; Fekonja et al., 2022; Ashourvan et al., 2020; Bernhardt et al., 2019). This flexibility ensures that digital twins can be tailored to address the unique challenges presented by different neurological conditions. To integrate the multidisciplinary approach into digital twin development, an optimal workflow should be established. This pipeline should begin with developing hypotheses to close gaps in knowledge or open new research fields, followed by data collection, incorporating neuroimaging, genomic data, neuropsychological assessments, and clinical outcomes and evaluation. Data processing should involve methods such as data harmonization and multimodal fusion to manage variability and ensure consistency across datasets. The integration phase should utilize both biological insights and philosophical perspectives to refine model parameters, ensuring that digital twins can be tailored to individual patients. Potential pitfalls include data privacy concerns, model overfitting, and the challenge of translating complex, multidisciplinary data into clinically actionable insights. However, with careful consideration of these factors, the aim of tailored therapy can be achieved, offering personalized treatment strategies that are informed by a comprehensive understanding of the patient’s unique pathology. For instance, while tumor models require additional components for simulating tumor growth and infiltration (Aerts et al., 2020), epilepsy models may need more detailed neural mass models to capture seizure dynamics (Jirsa et al., 2017).

In addition, as hinted above, the integration of digital twins in clinical practice raises significant concerns regarding data privacy, especially when utilizing protected medical records for training models. Compliance with regulations such as the General Data Protection Regulation (GDPR) in the European Union and the Health Insurance Portability and Accountability Act (HIPAA) in the United States is essential. Implementing robust data anonymization techniques and ensuring adherence to these regulations are critical to protect patient confidentiality while enabling the development of digital twins. To address these concerns, for example federated learning approaches allow model training on decentralized data, while differential privacy methods can be employed to protect individual patient data while still enabling useful model training (Kaissis et al., 2020).

Another challenge in developing digital twins in neuroscience is the integration of data from multiple sources. This requires advanced methodologies to manage variability and ensure consistency. Techniques such as data harmonization and federated learning can be employed to integrate diverse datasets, preserving the integrity of the models while accommodating variations in data quality and format. Multimodal fusion methods and Bayesian approaches like hierarchical modeling allow combining data with different noise characteristics and spatiotemporal resolutions, further enhancing the robustness and applicability of digital twin models in neuroscience (Friston et al., 2017; Calhoun and Sui, 2016).

## Pathology: its impact on the materiality of the brain

Due to the brain’s underlying network architecture, the effects of a tumor can extend beyond the vicinity of the tumor area. They can have potentially fatal impacts on cognition and often also on other functions such as motor systems. However, changed activity near the tumor may cause as well functional shifts, demonstrating the brain’s capacity for compensatory mechanisms. These adaptations, which involve changes in network connectivity and neural activity, are similar to responses observed in learning or after injury. They may result in the reallocation of specific functions to other brain areas. This process is termed *neuroplasticity.* Neuroplasticity refers to the brain’s ability to reorganize itself by forming new neural connections in response to injury, disease, learning or changes in the environment. This concept has been extensively studied in neuroscience and rehabilitation medicine, particularly in the context of plastic potentials and compensatory capabilities (Duffau, 2014; Rossi et al., 2021; Ius et al., 2011). In neurosurgery, intraoperative brain mapping has revealed different types of plasticity, including those that occur in response to tumor growth and resection, highlighting the dynamic nature of the brain’s response to pathology (Duffau, 2014). The inclusion of these studies provides a more comprehensive understanding of the brain’s adaptive and maladaptive responses, which are crucial for developing accurate digital twin models.

Recent studies on neuroplasticity demonstrate how the brain adapts to injury or structural changes (Piai et al., 2020; Hartwigsen and Saur, 2019; Herbet et al., 2016). These studies show that the brain can dynamically remodel its neuronal circuits to maintain functionality despite significant pathology. This occurs through molecular and cellular adaptations, such as the growth of new parts of the nerve cells and the cell’s firing strength, which are essential for the recovery or realignment of neurological functions. Further research shows that this plasticity goes beyond basic compensation and supports recovery through synaptic growth in injured areas (Pascual-Leone et al., 2005). These findings highlight neuroplasticity as an essential aspect of brain function that is critical for learning and adaptability (Diniz and Crestani, 2023; Price and Duman, 2020).

The extend of neuroplasticity can be illustrated through cases such as Mora Leeb’s: the entirety of her left hemisphere was removed from the 9 months old infant. Since the left hemisphere is considered crucial for language functions and controls right-sided motor functions, one would assume that a complete resection of the left part of the brain would result in a near-total loss of language functions and severe motor impairment. However, despite the resection of the left hemisphere, Mora Leeb was able to develop remarkable language and motor skills, demonstrating that the brain can reorganize and compensate, especially in response to early injury or neurosurgery in a patients early age. Like Leeb, another notable example is the case of Cameron Mott, who underwent hemispherectomy as a child to treat her severe epilepsy. Like Mora Leeb, Cameron experienced a remarkable recovery and adaptation after neurosurgery, demonstrating significant neuroplasticity that allowed her to regain much of her motor and cognitive function.

However, in contrast to potential longer-term compensatory mechanisms, there are as well historical examples where no compensatory mechanisms were observed due to the severe effects of instant brain injuries, like the aggressive impact of high-grade gliomas. Three cases that made history in behavioral neurology are often mentioned in that context: Phineas Gage, Monsieur Leborgne, and Henry Molaison. These cases are of crucial importance in neuroscience for understanding the limits of neuronal plasticity. They provide examples of our knowledge about the brain in relation to cerebral disconnection syndromes, their relation between brain architecture, network, and functioning, and the limits of compensatory mechanisms. Phineas Gage, an American railroad worker who lived in the 19*^th^* century, suffered a frontal lobe injury when a metal log propelled by a dynamite explosion at the railroad construction site pierced his head. While he survived this accident, the injury strongly changed his personality and social behavior, demonstrating that certain areas and related networks of the brain are critical to specific functions without apparent chance for compensation, especially due to sudden impact to the brain. Monsieur Leborgne experienced a stroke to his middle cerebral artery (MCA), which resulted in significant damage to his left frontal cortex and underlying white matter connectivity, leading to loss of speech production, indicating that area’s involvement in language function. In Henry Molaison, a bilateral resection of the medial temporal lobe was performed to treat his epilepsy, which unfortunately resulted in severe anterograde amnesia, demonstrating the role of these regions in relation to memory formation (Thiebaut de Schotten et al., 2015).

These three cases show the limitation in the brain’s capacity to adapt to trauma and demonstrate how certain types of lesions can lead to permanent impairments, providing insights into the brain’s connectivity and its relationship to function and cognition, but also regarding its adaptive capacity. These days, we can visualize and measure activities in the brain non-invasively using neuroimaging and measurement technology, as mentioned above. This also offers the possibility of modeling predictions, for example, and thus testing several variations before neurosurgery with digital twins.

Neuroscientific digital twins can play an important role in providing modeling capabilities to simulate complex dynamics of brain activity and dysfunction, including compensatory plasticity observed in response to such immediate pathologies as the ones just illustrated, such as accidental loss of parts of the brain. Based on that information, they can also serve to develop therapeutic strategies, for example in the context of preconditioning of certain brain areas prior to surgery to support compensatory neuronal plasticity. These virtual models can integrate neuronal activity and functional measurements from methods such as transcranial magnetic stimulation (TMS) and electroencephalography (EEG) that describe plasticity to predict how functions might behave in response to lesions. In addition, neuropsychological testing, when used in conjunction with TMS or EEG results prior to surgery, can measure and make comparable potential transient effects and other changes, increasing the accuracy and effectiveness of digital twin simulations in preoperative planning and ongoing treatment adjustments (Reisch et al., 2022; Wang et al., 2020; Rosenstock et al., 2017; Lefaucheur and Picht, 2016; Picht et al., 2016).

While the digital twin can be applied to examine and simulate phenomena such as plastic adaptabilities at different scales, as presented earlier, it can be thought of as well in different dimensions. Gliomas, for example, represent a common type of central nervous system tumors, originating from structural glia cells. They form a malignant tumor mass, which infiltrates the brain’s white matter over time. These tumors display prominent intrinsic plasticity themselves, by creating a flexible tumor ecosystem, rendering them resistant to therapies as evolutional mechanisms gave rise to specialized tumor cells. These plastic dynamics are understood to either present as the proliferation or selection of drug-resistant preexisting cellular conditions or evolve toward fit-for-recurrence phenotypes. As illustrated, zooming in to the cellular level reveals a form where tumor plasticity critically harms the brain, because it enables growth of a malignant cell mass resistant to targeted therapies. Understanding this *destructive* plasticity bottom-up, which means concluding consequences for the large-scale functional structure of the brain from observations at the microscopic level and addressing the brain’s multi-scale organization, can help to incorporate information about targets and potential therapy responses via digital twin models (D’Angelo and Jirsa, 2022). This understanding is further driven by basic research concerning cell markers and genetic phenomena. Results from these areas can further refine digital twins when merged with data from MRI and functional brain measures to pave the way for prediction of therapy trajectory in personalized medicine. In addition, they shape our understanding of the relationship between the cellular and functional aspects of the brain (Yabo et al., 2022; D’Angelo and Jirsa, 2022).

## The concept of destructive plasticity

The brain is subject to constant changes, induced by both external and internal factors, like learning, memory, neurodegenerative diseases, and pathologies such as stroke, tumors, or trauma. Complementary, as mentioned above, the brain is equipped with compensatory mechanisms to adapt, which further demonstrates the importance of plasticity. Philosophers like Catherine Malabou delve into the theoretical reflections of plasticity, raising broader questions about the nature of the brain and its impact on human identity (Malabou and Shread, 2018; Malabou, 2008). Digital twins can embody this philosophical exploration by providing a virtual platform on which both theoretical concepts and neurobiological frameworks, such as brain plasticity, can be observed and even tested in dynamic simulations. These digital representations bridge the gap between theory and practice, complementing discussions on ontological questions such as the nature of one person’s self, political implications, and deterministic necessities concerning the conceptualization of the brain and thus the nature of mankind (Malabou and Shread, 2018).

Malabou is a French philosopher. She is influenced by modern contemporary German philosophers, such as Kant, Hegel, and Heidegger. She is known for her treatise on brain plasticity and its implications for existential and identity-related philosophical questions. She developed a concept of plasticity which extends the incorporation of both adaptive and maladaptive processes. Moreover, Malabou argues that plasticity is understood mainly adaptive in various areas, constituting a continuous process of change, similar to sculpturing. Plasticity in a neurological context, for example, describes the adaptation of brain functions and structures to stimuli, changes due to internal or external conditions and suchlike (Malabou and Shread, 2018). In contrast to this widely accepted use of the term, she advocates to extend plasticity to also incorporate destructive, rather immediate occurrences like those found through trauma, strokes, or tumor and resulting resections, which often immediately sever cerebral connections (ibid.). She terms this *destructive plasticity*. Plasticity involves both construction as well as destruction of fundamental processes that are intertwined in the creation of new forms, including cells, larger organisms or brain functions and underlying structures. Both plastic processes are relevant in the formation of personality. Yet, Malabou claims that in disciplines like sciences, medicine, art and education only the adaptive part is accounted for as plasticity, when also destructive types can impact personality (Malabou and Miller, 2012). Thus, neurosurgical practice should indeed consider and, at best, even predict this type of destructive plasticity, often termed as well *maladaptive* plasticity (Johnson and Cohen, 2023; Jang, 2013). Therefore, the digital twinning practice we initially described needs to be adapted to address both types of plasticity.

A further aspect that is relevant to this practice is Malabou’s emphasis on the active, creative role of the brain in its own formation. She considers the brain not only as the recipient of changes that are inflicted on it. Rather, she emphasizes the importance of the brain as an active subject and initiator of chances and adaptations, as a constantly changing substance that is not simply affected by change but creates change itself. It can be understood as an antonym to rigidity, opposing the idea of a static or reacting organ in our biological system. On the contrary, the brain can be seen as an active creator without external stimuli, as with negative plastic processes that make it a generator of both positive and negative elements (Malabou and Shread, 2018, p.3).

However, the philosophical theory surrounding brain plasticity is not without controversy. While Malabou’s concept of destructive plasticity provides a unique lens through which to view the brain’s capacity for both adaptive and maladaptive changes, other philosophical perspectives offer alternative views on the nature of the brain and its relationship to identity and consciousness (Gallagher, 2017). For example, the enactive approach to cognitive science emphasizes the brain’s role in embodied action, suggesting that understanding brain function requires considering the organism’s interactions with its environment. This perspective, alongside Malabou’s, enriches the multidisciplinary approach of digital twin development by incorporating diverse philosophical insights into the modeling of brain dynamics.

This perspective on the brain evokes a power that shapes our perception of life and ourselves. From a position where the brain can initiate change, it creates the existential potential to have a certain influence on how we respond to the worldly dynamics around us. Malabou presents a dual pathway when she writes about the brain rather than the single track that it is only a product of our immediate environment, but also by inherent, internal dynamics and links the brain to the mind in a close relationship. This culminates in Malabou’s concept of semiotic materialism, where any form inherently carries the potential to transform and transcend its current state (Hogstad and Malabou, 2021). Semiotic materialism understands form beyond its immediate materiality and in context with its significance to represent human experience. This perspective emphasizes its interpretation in a scientific and social context, suggesting the brain is a form imbued with layers of meaning and possibilities. She opposed the thesis in which form is necessarily linked to presence and visibility according to her, a claim prominently pushed by Jaques Derrida (Hogstad and Malabou, 2021). Instead, she presents an existentialist position in which form, applied to the human brain, includes any prior and subsequent stages of the current one. Although Malabou’s work does not specifically ground plasticity in relation to brain tumors, her previously described concept of negative plasticity applies to this context here due to its immediate and radical characteristics and their maladaptive forms. This suggests that a person’s identity or a snapshot of a related brain composition already carries the next version, which in turn opens infinite possibilities, as indicated above. Applying this concept to neuroscience and neurological diseases, digital twins can enable calculations of how changes in the brain may alter its interconnectivity and cerebral functions based on it, as well as reveal the brain’s potential to adapt to change and in turn predicting variations in behavior. Here, plasticity is the determining component.

However, plasticity in Malabou’s sense can also eliminate any form, given or received. Therefore, Malabous’s philosophical approach to the role of plasticity in brain dynamics illustrates the importance of a comprehensive exchange between neurological phenomena and humanistic concepts, providing a framework to theorize digital twins in relation to pathology and inspiring questions about identity, agency, and subjectivity. While her political and social insights are not the focus here, it is worth noting that by further exploring concepts within the framework of a digital twin, she provides inspiration for approaching other philosophical viewpoints on the future of society and human interactions that could further advance the practice of digital twinning.

The plastic brain, capable of reciprocating changes after injury, can also provoke *explosive* dynamics, such as the growth of malignant cell tissue caused by early genetic coding defect. This is where plasticity takes on a rather unfavorable connotation. Malabou introduces her concept of *identité d’accident*, which delineates the consequence of such coincidental plasticity. A transformation of identity influenced by destruction becomes a possibility already inherent in its qualities. Malabou therefore challenges deterministic properties, as explosiveness does not follow a clear cause-and-effect scheme. In contrast to the aspects of positive plasticity, where the brain forms new connections to execute functions and successfully adapts to change, the explosiveness of malignancies in her philosophy, while already encoded in the structure as part of its definition, is rather aimless and indiscriminate. The out-of-control plasticity could manifest in anyone and at any time, often leaving those affected with an identity crisis (Malabou and Shread, 2018).

Drawing on Freud’s psychoanalytic understanding of the Psyche, which encompasses the mind and consciousness, and Spinoza’s monistic philosophy, which postulates a unity of body and spirit, Catherine Malabou underscores the necessity for an existential and vital configuration in neurobiology: an identity rooted in the experience of cerebral damage. She contends that neuroscience lacks a corresponding sense of destruction and loss when considering the central nervous system. It implies that this destructive plasticity corresponds with a metamorphosis, a discharge of the being from itself, remarkably, not in death but while alive. Thus, exploring negativity in the context of interpreting the brain could unveil new possibilities to understand it (Malabou and Shread, 2018).

According to Catherine Malabous’s philosophical position, the tendency toward destruction is embedded in everybody’s brain. While it remains speculative, the digital twin helps to reveal, also speculatively by using probabilities, the characteristics of the brain in cases where certain negative explosive qualities are prominently evident and active in patients suffering from a neuropathological disease. Therefore, by visualizing the brain and its changes during malignant development, the digital twin aids to introduce our above ideas into neuroscientific methodology. It demonstrates the interplay of either expression of plasticity by mapping the brain and its structural and functional configuration in the presence of a disruptive tumor. To construct a digital twin of a neurological patient’s brain, measurements are initially taken using TMS and EEG, which assess brain functions. Subsequently, tractography is employed to virtually delineate the brain’s white matter, revealing its connections to the cortex, the gray matter, and its network architecture. This mapping in digital space allows for a description of a brain tumor’s effects on the brain and its plastic abilities. Further research may even elucidate factors determining whether the tumor impedes self-transformation, causing substantial brain damage, or restrains the disruptive properties, which may cause the brain to compensate for the impaired functions.

Malabou’s work prompts questions about how the brain’s plasticity is altered or challenged by the presence of malignancy and how neurosurgery needs to be updated in this regard. As such, philosophy can help to navigate the complexity of neuroscience and critically reflect its findings, which could advance the narrative of the digital twin and the corresponding practical measures. By mentioning “All illnesses may be identical, but the sick are not[…].” Malabou opens a new window for valuable use and research regarding digital twinning (Malabou and Shread, 2018, p.68). Comprehending the development of a tumor is not sufficient in future medicine. Rather, the overall constitution of the patient is essential for effective treatment and deeper decoding of a pathology or condition. Since each patient’s brain is different, the effects on function and structure as well as the resulting response also vary, which can be assessed by digital twinning. It could therefore offer patients suffering from brain disorders information in which the pathology does not take away their identity, but rather demands a new one. This implies a paradigm that evolves toward Malabou’s understanding of the brain and goes beyond the distinction between healthy and an unhealthy brain. Thus, the connotations of right and wrong, healthy or defect, are defused and could potentially support patients themselves in therapy. However, we are not arguing that certain malignancies should not be considered as such. Nevertheless, digital twinning in neuroscience, alongside Malabou’s concepts, recognizes that e.g., brain tumors or injury can alter the brain’s structure and function. This transformative capacity of the brain illustrates both its adaptive and destructive potential. By understanding these dynamics, digital twins can not only support neurosurgical planning or neuroscientific findings but may as well facilitate the reintegration of patients into their communities. This concept demonstrates the interconnectedness of our cerebral structures and the brain’s capacity to reshape itself in response to injury. Further neuroscientific development makes the implementation of philosophical questions and assumptions essential to enrich neuro-oncological topics with ethics and ontology.

## Conclusion

In neuroscience, digital twins offer an approach to study the brain. These models can illustrate the complex relationships between micro-level and large-scale brain network dynamics and related functioning. Through the integration of multimodal data, such as MRI and neuropsychological assessments, they enable statistical modeling of tumor effects and compensatory mechanisms, which in turn further drives personalized therapy. By understanding both adaptive and destructive plasticity, inspired by Malabou’s philosophy, and from a bottom-up perspective, digital twins can not only inform therapeutic interventions, but also illuminate this topic from both a theoretical and practical perspective across different disciplines (Picht et al., 2021). Moreover, the distinction between adaptive and destructive forms illustrates the complexity associated with modeling a dynamic and fluid organ such as the brain. Furthermore, uncertainties associated with simulating a brain should be considered in the future development of digital twins as well as in relation to explainable AI. This provides another link that emphasizes theoretical speculation related to statistical uncertainty in digital twinning. Lastly, it is crucial to rigorously test the limitations of digital twin models to prevent misleading results. Acknowledging that models are only simplified representations and cannot fully capture the complexities of reality, it is essential to establish acceptable margins of error and understand the potential impacts of these limitations on clinical decisions. Continuous validation against real-world outcomes is vital to refine the models and minimize risks while increasing predictive accuracy. This ongoing process of validation and refinement ensures that digital twins in neuroscience remain reliable tools for both research and clinical applications, continually evolving to provide more accurate and useful insights into brain function and pathology.

## Data Availability

The original contributions presented in this study are included in this article/supplementary material, further inquiries can be directed to the corresponding author.
